# Supplementary material for: Phenylalanine Residues in the Active Site of CYP2E1 Participate in Determining the Binding Orientation and Metabolism-Dependent Genotoxicity of Aromatic Compounds
Source: Toxics. 2023 May 31;11(6):495. doi: 10.3390/toxics11060495 (PMC10302094; doi:10.3390/toxics11060495)
Supplement: Supplementary file 1 [file toxics-11-00495-s001.zip › toxics-2349424-supplementary.pdf]

Supplemental materials

Phenylalanine residues in the active site of CYP2E1 participate in determining the binding orientation and metabolism-dependent genotoxicity of aromatic compounds

Keqi Hu[1, 2, #], Hongwei Tu[3, #], Jiayi Xie[2, #], Zongying Yang[2], Huanzi Li[1], Yijing Chen[2], Yungang Liu[2,*]

[1] Department of Science and Education, Guangdong Second Provincial General Hospital, 466 Xingang Middle Road, Guangzhou 510317, China.
[2] Department of Toxicology, School of Public Health, Southern Medical University (Guangdong Provincial Key Laboratory of Tropical Disease Research), 1023 S. Shatai Road, Guangzhou 510515, China.
[3] Guangdong Provincial Center for Disease Control and Prevention, Qunxian Road, Panyu District, Guangzhou 511430, China.
[#] These authors contributed equally to this work.
* Correspondence: Yungang Liu, yungliu@126.com

Numbers of Tables: 4

Numbers of Figures: 1

Table S1. Molecular docking of 1-MP to human CYP2E1 under rigid and flexible settings with regard to the cavity volume, binding energy, and ligand-heme distance

PDB ID	Resolution	Rigid docking			PHEs flexible docking		
		Volume (Å3)	Binding energy score (kcal/mol)	Cα to Heme (Å)	Volume (Å3)	Binding energy score (kcal/mol)	Cα to Heme (Å)
3LC4	3.10	101.93	–8.24	4.4	101.59	–10.99	3.8
3KOH	2.90	110.40	–9.57	3.8	111.90	–10.71	4.0
3GPH	2.70	94.83	–9.30	3.2	82.77	–10.92	4.2
3E4E	2.60	69.22	–7.56	Outside*	78.35	–10.73	3.8
3T3Z	2.35	111.83	–8.82	10.4	95.69	–10.16	5.3
3E6I	2.20	83.38	–7.73	Outside*	86.01	–10.89	3.0
F298A	-	-	-	-	-	–10.62	4.7
F478A	-	-	-	-	-	–9.55	3.3

* Compound was bound to the enzyme at some position outside of the active site.

Table S2. Features of high rank tunnels for the binding of 1-MP to the active center of human CYP2E1

Compounds	Tunnels	Occurrence, %	Average bottleneck radius, Å	Maximum bottleneck radius, Å	Average tunnel length, Å
1-MP	2c	97.6	1.31 ± 0.16	1.71	21.44 ± 1.73
	2b	74.6	1.22 ± 0.16	1.72	30.12 ± 2.57

Table S3. The energy decomposition of non-PHE amino acid residues in the active sites of 1-MP-bound wild-type and mutated human CYP2E1

Residues	CYP2E1-WT kJ/mol	CYP2E1-F298A kJ/mol	CYP2E1-F478A kJ/mol	Energy Change
ILE115	−3.18 ± 1.31	−4.26 ± 0.71	−5.36 ± 0.89	↓
ASN206	−0.33 ± 0.10	−6.52 ± 0.98	−1.44 ± 0.32	↓
LEU210	−0.37 ± 0.16	0.16 ± 1.11	−2.01 ± 0.66	↓
ALA299	−4.16 ± 0.86	−3.17 ± 1.29	−2.69 ± 1.27	↑
GLU302	−2.62 ± 1.30	−3.23 ± 1.21	−0.94 ± 0.35	↑
THR303	−4.94 ± 1.12	−0.33 ± 0.09	−1.74 ± 1.72	↑
LEU363	−2.47 ± 1.04	−0.95 ± 0.20	−1.22 ± 0.64	↑
VAL364	−2.85 ± 1.70	−3.47 ± 0.70	−3.19 ± 0.91	↓
LEU368	−2.68 ± 0.58	1.25 ± 1.08	−6.20 ± 1.06	↓

Table S4. The molecular descriptors of PCB congeners as candidate factors influencing their binding to human CYP2E1 and orientation

PCB	MutaG (1-4)	CN	OP	MP	PP	DBES	DtF	L_HLG	L_EP	L_NP	P_HLG	P_EP	P_NP	FODw	FODm	FODp	MW	AHR	Kow	HLF
1	0	1	1	0	0	-7.00	3.2	7.67	1.18	1.66	7.36	1.22	1.86	0.102	0.106	0.098	188.65	28.5	4.47	7.1
2	0	1	0	1	0	-7.23	3.9	7.17	1.32	1.86	6.92	1.37	2.00	0.146	0.163	0.102	188.65	52.3	4.64	20.2
3	0	1	0	0	1	-5.40	10.0	7.06	1.29	1.99	7.00	1.27	2.08	0.108	0.114	0.127	188.65	38.5	4.61	35.3
4	1	2	2	0	0	-7.56	3.2	7.82	1.23	1.45	7.49	1.27	1.69	0.224	0.103	0.108	223.10	20.1	4.95	185.5
5	1	2	1	1	0	-7.71	3.9	7.74	1.31	1.49	7.28	1.32	1.77	0.165	0.144	0.120	223.10	29.3	5.02	141.4
6	1	2	1	1	0	-7.83	3.8	7.21	1.40	1.70	7.34	1.33	1.71	0.164	0.176	0.107	223.10	30.1	5.02	185.3
7	0	2	1	0	1	-7.32	3.9	7.41	1.29	1.69	7.24	1.31	1.80	0.231	0.168	0.112	223.10	26.3	5.13	42.4
8	1	2	1	0	1	-7.19	3.9	7.42	1.28	1.71	7.20	1.31	1.84	0.149	0.212	0.098	223.10	28.4	5.09	42.8
9	0	2	1	1	0	-7.85	3.7	7.41	1.33	1.65	7.17	1.32	1.87	0.136	0.160	0.121	223.10	30.6	5.13	239.1
10	1	2	2	0	0	-7.37	4.1	8.14	1.06	1.25	7.47	1.24	1.73	0.155	0.102	0.122	223.10	28.7	4.99	185.6
11	1	2	0	2	0	-7.49	4.6	7.03	1.47	1.76	6.99	1.45	1.83	0.164	0.172	0.104	223.10	40.5	5.28	28.5
12	1	2	0	1	1	-7.39	4.0	6.94	1.41	1.90	6.85	1.40	2.01	0.130	0.133	0.141	223.10	31.0	5.29	48.8
13	0	2	0	1	1	-5.83	10.0	6.74	1.49	1.97	6.89	1.42	1.95	0.162	0.160	0.133	223.10	30.3	5.16	63.5
14	0	2	0	2	0	-5.97	10.0	6.78	1.61	1.84	7.02	1.46	1.80	0.126	0.137	0.127	223.10	41.3	5.41	12.9
15	0	2	0	0	2	-7.33	8.5	6.74	1.42	2.06	6.80	1.36	2.10	0.168	0.134	0.139	223.10	20.2	5.26	12.5
18	1	3	2	1	0	-8.32	3.7	7.68	1.30	1.45	7.52	1.27	1.64	0.170	0.188	0.131	257.54	11.2	5.52	74.3
19	1	3	3	0	0	-7.65	3.0	8.04	1.18	1.29	7.48	1.18	1.81	0.118	0.130	0.114	257.54	11.2	5.48	315.2
20	1	3	1	2	0	-8.09	4.5	7.32	1.47	1.55	7.26	1.40	1.66	0.188	0.187	0.118	257.54	11.2	5.57	122.0
22	1	3	1	1	1	-7.85	4.5	7.28	1.39	1.65	7.16	1.36	1.81	0.179	0.168	0.115	257.54	11.3	5.44	144.2
27	1	3	2	1	0	-7.87	4.5	8.03	1.17	1.28	7.41	1.31	1.68	0.179	0.175	0.114	257.54	11.2	5.64	97.7
28	1	3	1	0	2	-7.76	5.8	7.14	1.39	1.75	7.02	1.39	1.86	0.148	0.145	0.127	257.54	11.0	5.62	75.9
32	1	3	2	0	1	-7.26	3.7	7.98	1.18	1.33	7.41	1.32	1.65	0.165	0.153	0.116	257.54	1.12	5.75	274.2
40	0	4	2	2	0	-8.56	4.5	7.91	1.34	1.20	7.45	1.29	1.66	0.159	0.155	0.133	291.98	7.68	6.04	102.6
46	0	4	3	1	0	-8.35	4.4	7.96	1.31	1.21	7.44	1.29	1.76	0.147	0.182	0.132	291.98	7.64	6.18	153.5
52	1	4	2	2	0	-6.37	10.0	7.56	1.42	1.39	7.44	1.36	1.59	0.207	0.190	0.162	291.98	7.92	6.18	262.4
54	0	4	4	0	0	-5.60	10.0	8.06	1.24	1.22	7.54	1.20	1.67	0.171	0.148	0.134	291.98	7.44	5.94	153.3

56	1	4	1	2	1	-8.27	4.5	7.37	1.45	1.48	7.16	1.48	1.63	0.197	0.198	0.134	291.98	8.71	6.17	295.5
66	1	4	1	1	2	-8.60	5.6	7.09	1.49	1.64	7.02	1.47	1.75	0.243	0.177	0.139	291.98	9.12	6.11	119.6
74	1	4	2	1	1	-8.11	3.8	7.71	1.36	1.37	7.51	1.32	1.66	0.169	0.201	0.141	291.98	9.13	6.66	346.9
77	0	4	0	2	2	-8.62	8.3	6.55	1.68	1.87	6.71	1.56	1.90	0.253	0.250	0.171	291.98	10.1	6.62	22.7
81	0	4	0	2	2	-6.39	10.0	6.51	1.70	1.88	6.72	1.57	1.87	0.189	0.200	0.176	291.98	10.1	6.15	48.8
105	1	5	1	2	2	-8.50	5.3	7.08	1.57	1.55	7.02	1.54	1.66	0.283	0.245	0.160	326.42	6.49	6.79	115.7
118	1	5	1	2	2	-8.67	5.4	7.40	1.54	1.31	6.89	1.53	1.75	0.250	0.243	0.164	326.42	6.41	7.11	154.7
126	0	5	0	3	2	-8.71	7.3	6.50	1.80	1.75	6.64	1.66	1.81	0.274	0.273	0.204	326.42	6.63	6.56	250.4

MutaG, human CYP2E1-activated mutagenicity of a PCB congener (1-4); CN, the number of Cl-substitutions; OP, the number of ortho-Cl-substitutions; MP, the number of meta-Cl-substitutions; PP, the number of para-Cl-substitutions; DBES, binding energy score obtained from molecular docking with PHE478 being set flexible; DtF, the distance from SOM to Fe ion in the heme; L_HLG, the HOMO-LUMO gap of ligand; L_EP, the electrophilicity of ligand; L_NP, the nucleophilicity of ligand; P_HLG, the HOMO-LUMO gap of ligand-PHE complex; P_EP, the electrophilicity of ligand-PHE complex; P_NP, the nucleophilicity of ligand-PHE complex; FODw, the FOD value of ligand bound to the active site of human CYP2E1; FODm, the FOD value of ligand bound to the active site of F478A mutant; FODp, the FOD value of ligand; Mass, the molecular weight; AHR, atmospheric hydroxylation rate (cm^3/molecule*sec); Kow, LogKow: Octanol-Water partition coefficient; HLF, the half-life (by biotransformation and elimination) in fish.

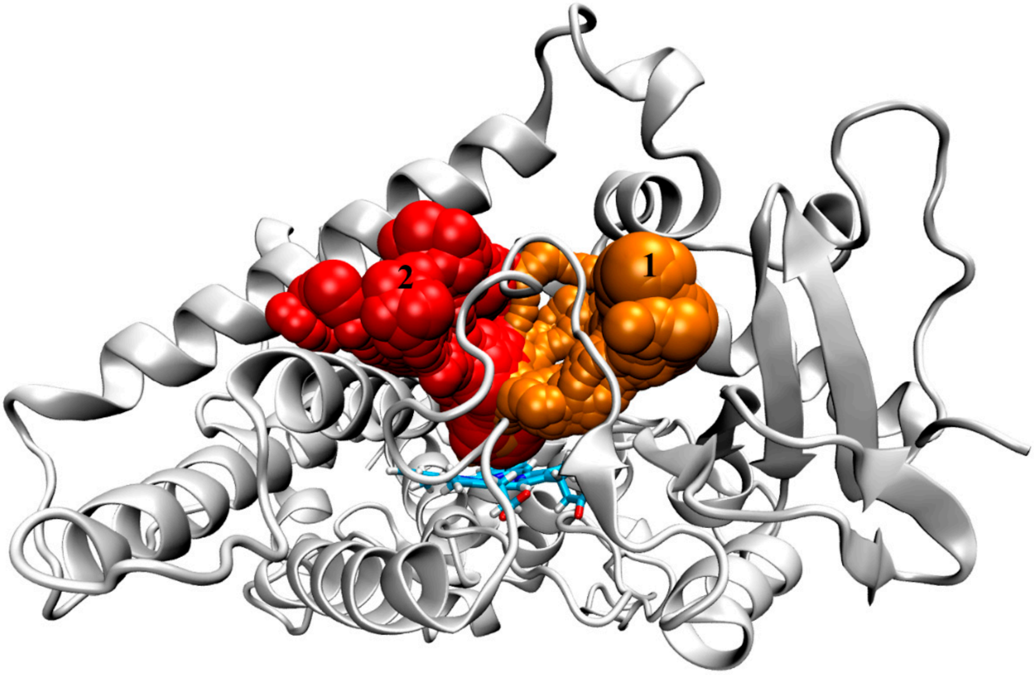

Figure S1. Two major tunnels for ligands entering the active site in human CYP2E1 Tunnel 1 indicates the 2b tunnel, which is controlled by PHE478; Tunnel 2 indicates the 2c tunnel, as controlled by PHE298.

References

1. Liu Y, Hu K, Jia H, Jin G, Glatt H, Jiang H. Potent mutagenicity of some non-planar tri- and tetrachlorinated biphenyls in mammalian cells, human CYP2E1 being a major activating enzyme. Arch Toxicol. 2017;91(7):2663-76.

2. Zhang C, Lai Y, Jin G, Glatt H, Wei Q, Liu Y. Human CYP2E1-dependent mutagenicity of mono- and dichlorobiphenyls in Chinese hamster (V79)-derived cells. Chemosphere. 2016;144:1908-15.

3. Chen Y, Zhu N, Luo Y, Hu K, Liu Y. Featured structure-activity relationships for some tri- and tetrachlorobiphenyls in human CYP2E1-activated mutagenicity - Impact of the extent of ortho-chlorination. Chemosphere. 2018;210:467-75.

4. Hu K, Yu H, Li Z, Jin G, Jia H, Song M, et al. Human CYP2E1-activated mutagenicity of dioxin-like PCBs 105 and 118-Experimental data consistent with molecular docking results. Toxicology. 2020;437:152438.
